# Survival Insights in Locally Advanced Gallbladder Cancer: The Role of ^18^ F-FDG PET/CT

**DOI:** 10.1055/s-0046-1824336

**Published:** 2026-06-04

**Authors:** Shantanu Pande, Zaeba Nayeem, Nihit Mhatre, Abhishek Gawande, Jyoti Ranjan Swain

**Affiliations:** 1 Department of Nuclear Medicine and Molecular Imaging, All India Institutes of Medical Sciences, Nagpur, Maharashtra, India; 2 Department of Nuclear Medicine, M. S. Ramaiah University of Applied Sciences, Ramaiah Medical College, Bangalore, Karnataka, India; 3 Department of Nuclear Medicine, Jupiter Hospital, Pune, Maharashtra, India; 4 Department of Nuclear Medicine, All India Institutes of Medical Sciences, Nagpur, Maharashtra, India; 5 Department of Surgical Oncology, Acharya Harihar Cancer Institute, Cuttack, Odisha, India

**Keywords:** FDG PET/CT, locally advanced GBC, metabolic tumor volume, prognosis, total lesion glycolysis

## Abstract

**Background:**

Gallbladder carcinoma (GBC) is an aggressive malignancy with a poor prognosis, often diagnosed at a locally advanced stage. Accurate risk stratification is crucial for optimizing treatment, yet conventional imaging and staging systems have limitations. This study aimed to evaluate the prognostic value of metabolic and volumetric parameters from ^18^ F-fluorodeoxyglucose positron emission tomography/computed tomography ( ^18^ F-FDG PET/CT) in patients with locally advanced GBC.

**Methods:**

This retrospective study included 32 patients with biopsy-confirmed, locally advanced GBC who underwent baseline ^18^ F-FDG PET/CT. The standard uptake values (SUVmax, SUVmean), metabolic tumor volume (MTV), and total lesion glycolysis (TLG) were measured for both primary tumors and nodal metastases. A univariate Cox proportional hazards model was used to assess the association between these volumetric metabolic PET parameters and progression-free survival (PFS) and overall survival (OS). The multivariate Cox model was used to test the independence of significant univariate prognostic factors.

**Results:**

In the univariate analysis of volumetric metabolic PET parameters of primary tumors, higher pSUVmean (hazard ratio [HR]: 1.39, *p* = 0.04), pTLG2.5 (per 100 units; HR: 1.01, *p* = 0.02), pMTV40 (HR: 1.00, *p* = 0.02), and pTLG40 (per 100 units; HR: 1.09, *p* = 0.01) were all significantly associated with worse OS. Similar significant associations were found for PFS. Notably, the conventional metric of pSUVmax was not a significant predictor of either PFS ( *p* = 0.11) or OS ( *p* = 0.25). On multivariate analysis, pTLG40 remained an independent predictor of survival. Furthermore, none of the volumetric metabolic PET parameters derived from nodal metastases showed a significant association with survival outcomes.

**Conclusion:**

Volumetric metabolic PET parameters, particularly TLG and MTV, which reflect the total metabolic burden of the primary tumor, may offer greater prognostic utility in locally advanced GBC compared with the conventional SUVmax. These parameters should be considered for integration into prognostic models to enhance patient risk stratification and guide personalized therapeutic strategies.

## Introduction

Gallbladder carcinoma (GBC) is an aggressive malignancy of the biliary tract with multiple etiological factors contributing to its development. 1 Due to its nonspecific clinical presentation, GBC is often diagnosed at an advanced stage, resulting in poor prognosis and low 5-year survival rates. 2 The disease is characterized by local invasion, extensive regional nodal involvement, and early dissemination, making timely and accurate stratification of patients crucial for optimizing treatment decisions. Surgery with negative resection margins remains the only curative approach; however, only approximately 10% of patients qualify for surgical intervention. 3 4 5 6

The TNM staging system, established by the International Union Against Cancer and the American Joint Committee on Cancer, is widely used for classifying GBC based on depth of tumor invasion and involvement of lymph nodes with distant metastases. 7 Despite thorough preoperative assessments, up to one-third of biliary malignancy patients undergo unnecessary laparotomy, highlighting the limitations of anatomical imaging in accurate patient stratification. 7

^18^ F-fluorodeoxyglucose positron emission tomography/computed tomography ( ^18^ F-FDG PET/CT) integrates functional and anatomical imaging, making it indispensable for cancer detection staging, restaging, and treatment response evaluation. 8 9 By assessing glucose metabolism, ^18^ F-FDG PET/CT provides insights into tumor activity, with SUVmax (maximum standardized uptake value) being the most commonly used semiquantitative parameter. While SUVmax has demonstrated prognostic value, it represents only a single voxel measurement and does not capture the entire metabolic activity of a tumor. 8 9

To overcome these limitations, metabolic tumor volume (MTV) and total lesion glycolysis (TLG) were evaluated as more comprehensive metabolic markers. MTV quantifies the active tumor volume in cubic centimeters, using either a 40% threshold of SUVmax or a fixed SUV cutoff of 2.5. TLG, a volumetric measure of glycolytic activity, is derived by multiplying MTV by SUVmean. 10 11 Studies have demonstrated the prognostic significance of MTV and TLG in various cancers, including esophageal, head and neck, and pancreatic cancers, with findings suggesting that these markers may provide superior prognostic value compared with SUVmax alone. 10 11 However, research evaluating their role in GBC remains limited.

This study aims to assess the prognostic utility of MTV and TLG derived from ^18^ F-FDG PET/CT in locally advanced GBC, exploring their correlation with survival outcomes and their potential to improve patient stratification beyond conventional anatomical imaging.

## Materials and Methods

### Study Design and Population

This retrospective, observational study included biopsy-confirmed cases of locally advanced GBC who underwent baseline ^18^ F-FDG PET/CECT (contrast-enhanced CT) imaging between September 2018 and May 2020. A total of 32 patients, aged 30 to 70 years, were enrolled. Eligible patients had T3 or T4 gallbladder lesions with local invasion, liver infiltration, bile duct obstruction, and involvement of adjacent gastrointestinal structures, including the pyloric region, duodenum, stomach, and hepatic flexure, along with regional lymph node metastases affecting the hepatic artery and hepatoduodenal ligament. Additionally, incidental GBC cases with locally invasive residual or recurrent disease in the gallbladder fossa were considered, although a significant number were evaluated only for initial staging.

Patients were excluded if they had metastatic or localized GBC, insufficient medical records, blood glucose levels >200 mg/dl, synchronous primary malignancies, or had previously received cancer-directed systemic therapy. Institutional Ethics Committee approval was obtained for this study, ensuring adherence to ethical research guidelines.

### 18F-FDG PET/CT Imaging and Analysis

All patients fasted for a minimum of 6 hours before undergoing ^18^ F-FDG PET/CT imaging. An intravenous dose of 5 MBq/kg body weight of FDG was administered, and imaging was performed 45 to 60 minutes post-injection using a combined PET/CT scanner system with a 16-slice CT scanner (Discovery IQ, GE Healthcare, Milwaukee, Wisconsin, United States). Patients were instructed to evacuate their urinary bladder before scanning to minimize physiological uptake interference.

CECT was performed unless contraindicated, using an iodinated contrast medium administered intravenously. The CT acquisition started at the base of the skull and extended to the mid-thigh (140 kVp, automatic mA, and 3.75 mm slice thickness). Immediately after, PET emission scans covering the same body region were acquired. Attenuation-corrected PET images were reconstructed using CT data via an Ordered Subsets Expectation Maximization (OSEM) algorithm, and image analysis was conducted using Advantage Workstation Software (GE Healthcare).

### Image Interpretation and Metabolic Parameter Analysis

All ^18^ F-FDG PET/CT studies were reviewed on an advanced workstation by two board-certified nuclear medicine physicians (each with more than 5 years of PET experience), blinded to clinical outcomes. Primary-tumor volumes of interest (VOIs) were outlined semi-automatically using an absolute SUV threshold of 2.5 (MTV _2_ . _5_ ) and a relative threshold of 40% of each lesion's SUVmax (MTV _40_ ). TLG was calculated as MTV × SUVmean for both threshold methods (TLG _2_ . _5_ , TLG _40_ ). SUVmax and SUVmean were recorded from the hottest 3D VOI pixel and the corresponding VOI, respectively. Lymph nodes were classified as involved if focal FDG uptake exceeded background mediastinal blood-pool activity and correlated with CT morphology; nodal MTV and TLG were measured using the same 2.5 SUV and 40% SUVmax thresholds.

### Treatment and Clinical Follow-Up

All the patients were treated with chemotherapy. For follow-up and treatment response evaluation, abdominal CT scans were acquired and in six patients, ^18^ F-FDG PET/CT was available. If follow-up imaging is not available, then clinical records of the last follow-up visit were considered. The treatment response was evaluated based on RECIST 1.1 or PERCIST criteria. The radiological findings were considered stable only when follow-up imaging was performed at 6 months. A minimum follow-up of 4 months was required. Disease status (progression or stable disease) was determined at each visit by combining imaging and clinical data, and the dates of confirmed progression or death were recorded. Patients without incidents have been excluded as of the date of the last follow-up.

### Statistical Analysis

Statistical analyses were conducted in R (v4.x) using the survival and survminer packages. Continuous variables (PET parameters, follow-up time) were described as mean ± standard deviation (SD) and median (interquartile range [IQR]), and categorical outcomes as counts. Progression-free survival (PFS) and overall survival (OS) were calculated from the date of PET/CT to the date of progression, death, or last follow-up and were censored otherwise. Univariate Cox proportional hazards models estimated hazard ratios (HRs) per unit increase in SUVmax, SUVmean, MTV _2_ . _5_ , TLG _2_ . _5_ , MTV _40_ , and TLG _40_ for PFS and OS; nodal parameters were analyzed similarly in the subset with FDG-avid nodes. A multivariate Cox model was then applied to assess the independence of the most significant prognostic variables identified in the univariate analysis. A two-tailed *p* <0.05 was deemed statistically significant consistently. Survival curves were estimated using the Kaplan–Meier method to visualize the prognostic value of metabolic tumor burden. Differences in PFS between groups were assessed using the log-rank test. For this analysis, the cohort was dichotomized into “High” and “Low” groups based on the median value of the significant predictor (pTLG40).

## Results

### Patient Characteristics and Follow-Up

We identified 32 patients who underwent staging ^18^ F-FDG PET/CT at our institution between October 2018 and June 2020. Scan dates were paired with either the date of progression, death, or last clinical follow-up to calculate individual follow-up times. Patients were classified into three outcome groups: death ( *n* = 13), documented progression ( *n* = 14), or no progression at last follow-up ( *n* = 5).

Follow-up durations for the entire cohort and by outcome subgroup are summarized in Table 1 . For all 32 patients, the mean follow-up time was 265.6 days (SD ± 262.4 days), with a median of 200.5 days (IQR: 84–285 days). As expected, patients who died had the shortest follow-up, while the five patients without progression at the end of the study period had the longest duration of follow-up.

**Table 1:** Summary of the entire cohort ( *N* = 32), the follow-up durations of the cohort, and its subgroups based on outcome (in days)

	*N*	Mean	SD
All patients	32	265.6	262.4
Death	13	148.5	96
No progression	5	615.2	379.9
Progression	14	253.7	223.3

Abbreviation: SD, standard deviation.

### Volumetric metabolic PET Parameter Analysis

The volumetric metabolic PET parameters derived from ^18^ F-FDG PET/CT for the primary tumor and nodal regions are summarized in Table 2 . The data reveal a wide range of metabolic activity and tumor burden across the cohort, particularly for the volumetric metabolic parameters such as pMTV and pTLG, indicating significant heterogeneity among the patient population.

**Table 2:** Summary of the volumetric metabolic PET parameters derived from ^18^ F-FDG PET/CT for the primary tumor and lymph nodes

Parameters	*N*	Mean	SD
pSUVmax	32	12.05	7.48
pSUVmean	32	4.41	1.74
pMTV2.5 (mL)	32	846.9	820.0
pTLG2.5	32	3,097.43	3,704.99
pMTV40 (mL)	32	479.8	383.2
pTLG40	32	682.02	1,000.99
nSUVmax	24	7.12	4.01
nSUVmean	24	3.42	0.75
nMTV2.5 (mL)	24	7.58	4.50
nTLG2.5	24	165.71	272.78
nMTV40 (mL)	24	3.94	2.30
nTLG40	24	374.32	651.99

Abbreviations: CT, computed tomography; PET, positron emission tomography; SD, standard deviation.

Note: pSUVmax, maximum standardized uptake value of primary tumor; pSUVmean, mean standardized uptake value of primary tumor; pMTV2.5, metabolic tumor volume of primary tumor with fixed SUV cut-off of 2.5; pTLG2.5, total lesional glycolysis of primary tumor with fixed SUV cut-off of 2.5; pMTV40, metabolic tumor volume of primary tumor with 40% threshold of SUVmax; pTLG40, total lesional glycolysis of primary tumor with 40% threshold of SUVmax; nSUVmax, maximum standardized uptake value of lymph node; nSUVmean, mean standardized uptake value of lymph node; nMTV2.5, metabolic tumor volume of lymph node with fixed SUV cut-off of 2.5; nTLG2.5, total lesional glycolysis of lymph node with fixed SUV cut-off of 2.5; nMTV40, metabolic tumor volume of lymph node with 40% threshold of SUVmax; nTLG40, total lesional glycolysis of lymph node with 40% threshold of SUVmax.

### Univariate Survival Analysis

The association between each volumetric metabolic PET parameter and patients' survival outcome was examined using univariate Cox proportional hazard models. The results are presented in Tables 3 , 4 , and 5 .

**Table 3:** Association between volumetric metabolic PET parameters of primary tumor and progression-free survival using univariate Cox proportional hazard models

Parameters	HR per unit (95% CI)	*p* -Value
pSUVmax	1.05 (0.99–1.12)	0.11
pSUVmean	1.45 (1.09–1.94)	0.01 a
pMTV2.5	1.00 (1.00–1.00)	0.06
pTLG2.5 (per 100 units)	1.01 (1.00–1.02)	<0.01 a
pMTV40 (per 100 units)	1.24 (1.06–1.45)	0.01 a
pTLG40 (per 100 units)	1.09 (1.02–1.16)	0.01 a

Abbreviations: CI, confidence interval; PET, positron emission tomography.

a Statistically significant at *p* < 0.05.

**Table 4:** Association between volumetric metabolic PET parameters of primary tumor and overall survival using univariate Cox proportional hazard models

Parameters	HR per unit (95% CI)	p-value
pSUVmax	1.04 (0.97–1.11)	0.25
pSUVmean	1.39 (1.01–1.91)	0.04 a
pMTV2.5	1.00 (1.00–1.00)	0.10
pTLG2.5 (per 100 units)	1.01 (1.00–1.02)	0.02 a
pMTV40 (per 100 units)	1.22 (1.04–1.43)	0.02 a
pTLG40 (per 100 units)	1.09 (1.02–1.16)	0.01 a

Abbreviations: CI, confidence interval; PET, positron emission tomography.

a Statistically significant at *p* < 0.05.

**Table 5:** Association between volumetric metabolic PET parameters of lymph nodes and patients' survival outcome (PFS and OS) using univariate Cox proportional hazard models

Parameters	PFS HR (95% CI)	PFS p-value	OS HR (95% CI)	OS *p* -value
nSUVmax	1.04 (0.92–1.18)	0.51	1.02 (0.91–1.15)	0.70
nSUVmean	1.03 (0.59–1.78)	0.93	0.90 (0.51–1.58)	0.71
nMTV2.5	1.00 (0.99–1.01)	0.93	1.00 (0.99–1.01)	0.81
nTLG2.5	1.00 (1.00–1.01)	0.38	1.00 (0.99–1.00)	0.33
nMTV40	1.00 (0.98–1.02)	0.89	1.00 (0.99–1.01)	0.78
nTLG40	1.00 (1.00–1.00)	0.45	1.00 (1.00–1.00)	0.42

Abbreviations: CI, confidence interval; HR, hazard ratio; OS, overall survival; PFS, progression-free survival.

Note: Statistically significant at *p* < 0.05. Nodal volumetric metabolic PET parameters ( *N* = 24).

For PFS, several volumetric metabolic PET parameters of the primary tumor were significant predictors of outcome ( Table 3 ). Higher values of pSUVmean (HR: 1.45, *p* = 0.01), pTLG _2_ . _5_ ( *p* < 0.01), pMTV _40_ ( *p* = 0.01), and pTLG _40_ ( *p* < 0.01) were all significantly associated with an increased risk of disease progression.

A similar pattern was observed for OS, as shown in Table 4 . Once again, pSUVmean (HR: 1.39, *p* = 0.04), pTLG _2_ . _5_ ( *p* = 0.02), pMTV _40_ ( *p* = 0.02), and pTLG _40_ ( *p* = 0.01) were all significantly associated with an increased risk of death. Notably, the conventional PET parameter of pSUVmax did not show a statistically significant association with either PFS ( *p* = 0.11) or OS ( *p* = 0.25).

In striking contrast to the primary tumor findings, none of the nodal volumetric metabolic PET parameters showed a significant association with either PFS or OS in this cohort ( Table 5 ).

### Multivariate Survival Analysis

To determine the most robust prognostic biomarker, a multivariate analysis was performed for OS, including the two strongest predictors from the univariate analysis. In this model, pTLG _40_ remained a powerful and independent predictor of death (adjusted HR per 100 units: 1.08; 95% confidence interval: 1.01–1.15; *p* = 0.02), while pSUVmean lost its statistical significance. However, this multivariate analysis has a small sample size and a limited number of events, hence it must be interpreted cautiously. The results are summarized in Table 6 .

**Table 6:** Multivariate analysis for overall survival

Parameters	Adjusted HR (95% CI)	*p* -Value
pTLG40 (per 100 units)	1.08 (1.01–1.15)	0.02 a
pSUVmean	1.25 (0.90–1.74)	0.18

Abbreviation: CI, confidence interval.

a Statistically significant at *p* < 0.05.

**Fig. 1:**
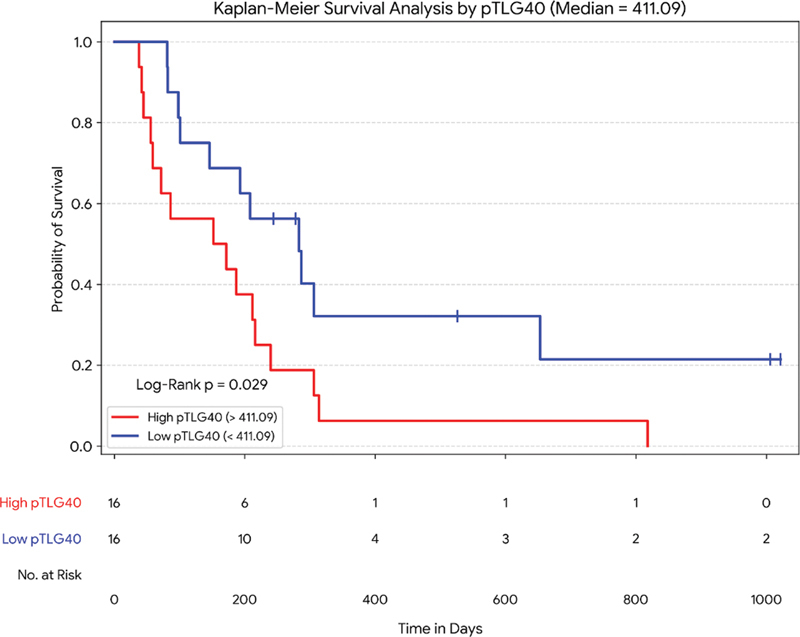
Kaplan–Meier estimates of progression-free survival (PFS) stratified by metabolic tumor volume (pTLG40). The cohort ( *n* = 32) was dichotomized by the median pTLG40 value of 411.09. The High pTLG40 group (red line) demonstrated significantly inferior survival compared with the Low pTLG40 group (blue line), with a median survival of 152 days versus 283 days ( *p* = 0.029, log-rank test). Vertical tick marks represent censored patients (those with no progression at last follow-up). The table below the x-axis indicates the number of patients at risk at each time interval.

To further evaluate the clinical impact of metabolic tumor burden, we performed a Kaplan–Meier survival analysis stratified by pTLG40. Patients were divided into high (>411.09) and low (≤411.09) burden groups. The analysis revealed a significant divergence in clinical outcomes ( Fig. 1 ). Patients in the High pTLG40 group demonstrated significantly inferior PFS compared with the Low pTLG40 group (log-rank *p* = 0.029). The median survival time was nearly double for patients with lower metabolic burden (283 days) compared with those with high burden (152 days), highlighting the ability of pTLG40 to identify patients at high risk of early progression or mortality. However, this must be interpreted carefully, as the median-based splitting pTLG40 with a small sample size and lack of adjustment for confounders may limit its relevance.

## Discussion

GBC is an aggressive malignancy with high glycolytic activity, showing strong FDG uptake on ^18^ F-FDG PET/CT, which is effective for staging, restaging, and detecting metastases. 12 13

While SUVmax correlates with prognosis, it reflects only peak activity, not total tumor burden. To address this, volumetric metabolic PET parameters—MTV and TLG—provide a more thorough evaluation. These parameters better capture tumor heterogeneity and have shown prognostic value across cancers, though studies in locally advanced GBC remain limited.

This study demonstrates that in patients with locally advanced GBC, volumetric metabolic parameters derived from ^18^ F-FDG PET/CT are likely strong predictors of survival and potentially perform better than the conventional parameter of SUVmax. However, further analysis by comparative metrics with a large sample size is required to establish its superiority. Our principal finding is that volumetric metabolic parameters reflecting the total metabolic burden of the primary tumor—specifically TLG, MTV, and even the mean standardized uptake value (SUVmean)—are associated with both progression-free and OS. In striking contrast, we found that neither SUVmax of the primary tumor nor any volumetric metabolic parameters from nodal disease held significant prognostic value in this cohort. Furthermore, on multivariate analysis, the prognostic power of pTLG _40_ was independent of pSUVmean, identifying it as the most promising metabolic biomarker in our study.

Our results support the growing consensus across oncology that volumetric metabolic PET parameters provide a more comprehensive and biologically relevant assessment of tumor aggressiveness than single-voxel measurements like SUVmax. 8 9 While SUVmax identifies the single most metabolically active point within a tumor, it fails to capture the lesion's heterogeneity or its overall metabolic footprint. Volumetric metabolic PET parameters like MTV and TLG, however, integrate both the size and the glycolytic activity of the entire tumor, offering a more complete picture of the disease burden. The finding that a higher pTLG _40_ increased the risk of progression and death—with every 100-unit increase elevating the HR by 9%—likely represents a potential, quantifiable measure of this risk. This aligns with findings in other gastrointestinal and head and neck cancers, where TLG and MTV have proven to be superior prognosticators, 10 11 and our study now firmly extends this principle to locally advanced GBC.

The biological rationale for these findings is clear. A high TLG represents a tumor that is not only large but also has high glucose consumption, a surrogate for increased cellular proliferation, hypoxia, and aggressive biological behavior. The independent prognostic significance of pSUVmean also favorably suggests that the *average* metabolic rate across the entire tumor is more indicative of patient outcome than the single hottest spot. Perhaps the most surprising finding was the lack of prognostic significance for any nodal volumetric metabolic parameter. This suggests that in the setting of locally advanced GBC, the biological aggressiveness and metabolic burden of the primary tumor itself may be the overwhelming driver of prognosis, potentially eclipsing the additional information provided by the metabolic status of regional nodes.

The clinical implications of these findings are potentially substantial. Accurate risk stratification is paramount in GBC to guide difficult treatment decisions, such as determining suitability for aggressive surgery versus neoadjuvant or palliative approaches. 7 Our data suggest that incorporating volumetric metabolic PET parameters into the initial staging process could potentially refine this risk assessment. For example, a patient with a high pTLG _40_ could be identified as being at very high risk for recurrence and death, perhaps making them a better candidate for neoadjuvant systemic therapy to treat a micrometastatic disease before considering surgery. Conversely, a patient with a low-burden, low-activity tumor might be prioritized for an upfront surgical approach. This provides a clear pathway toward more personalized, risk-adapted treatment strategies based on the unique metabolic signature of each patient's tumor. The separation of survival curves between high and low pTLG40 groups suggests that the total glycolytic volume may serve as a more clinically relevant biomarker for risk stratification in gallbladder cancer, potentially guiding the intensification of neoadjuvant therapies for high-risk patients.

This study has limitations. The retrospective design, single-center cohort, and modest sample size ( *N* = 32) may limit the generalizability of our findings. Because testing for multiple PET parameters was conducted across two different survival outcomes, there is an inherent risk of α error inflation. The lack of statistical significance for nodal parameters, for instance, could be due to the study being underpowered to detect a more subtle effect in the 24 patients with FDG-avid nodes. Furthermore, the optimal method for defining volumetric metabolic parameters (e.g., absolute vs. relative thresholds) requires further standardization across institutions. Depending upon the segmentation methods used in locally advanced GBC, the volumetric metabolic parameters may be underestimated or overestimated. Adenocarcinoma is the most common histopathological variant; however, other variants like papillary carcinoma, mucinous, squamous, and adenosquamous carcinomas were reported, and this variation in histopathology was not considered in multivariate analysis. Patients included ranged from 30 to 70 years of age, but this factor was not evaluated. Bilirubin levels and systemic inflammatory markers (i.e., C-reactive protein) were not included. Bilirubin levels and C-reactive protein levels are commonly available laboratory tests and their comparison value is limited due to various laboratory-associated factors. Serum CA 19–9 levels were not recorded; heterogeneity of follow-up methods and limited use of PET-based response criteria represent important limitations of our study. Due to these unadjusted key confounders and the potentially underpowered multivariate analysis, our results should be framed as one of the preliminary studies. Hence, further studies are required to validate prognostic values of volumetric metabolic parameters of 18F-FDG PET/CT in GBC.

## Conclusion

In conclusion, our study provides investigative evidence that volumetric metabolic PET parameters, particularly TLG and MTV may offer greater prognostic utility compared to SUVmax for prognosticating survival in patients with locally advanced GBC. These parameters, which holistically capture the tumor's metabolic burden, could be considered for integration into prognostic models to improve patient risk stratification and help guide critical therapeutic decisions in this challenging disease.
